# Synthetic Compounds with 2-Amino-1,3,4-Thiadiazole Moiety Against Viral Infections

**DOI:** 10.3390/molecules25040942

**Published:** 2020-02-19

**Authors:** Georgeta Serban

**Affiliations:** Pharmaceutical Chemistry Department, Faculty of Medicine and Pharmacy, University of Oradea, 29 Nicolae Jiga, 410028 Oradea, Romania; getaserban_2000@yahoo.com; Tel.: +4-0756-276-377

**Keywords:** 2-amino-1,3,4-thiadiazole, viral infections, antiviral agents, drug resistance, inhibitory concentration, mechanism of action

## Abstract

Viral infections have resulted in millions of victims in human history. Although great efforts have been made to find effective medication, there are still no drugs that truly cure viral infections. There are currently approximately 90 drugs approved for the treatment of human viral infections. As resistance toward available antiviral drugs has become a global threat to health, there is an intrinsic need to identify new scaffolds that are useful in discovering innovative, less toxic and highly active antiviral agents. 1,3,4-Thiadiazole derivatives have been extensively studied due to their pharmacological profile, physicochemical and pharmacokinetic properties. This review provides an overview of the various synthetic compounds containing the 2-amino-1,3,4-thiadiazole moiety that has been evaluated for antiviral activity against several viral strains and could be considered possible prototypes for the development of new antiviral drugs.

## 1. Human Viral Infections

Viruses are the smallest among all self-replicating organisms and yet they are the etiological agents of many difficult to treat diseases in human populations [1]. There are broad types of human infections caused by viruses, such as respiratory infections (common cold, Influenza), digestive infections (viral gastroenteritis), central nervous system infections (viral meningitis, viral encephalitis), skin or mucosal infections (herpes, measles, mumps, smallpox and rubella), hepatic infections (hepatitis A, B, C, E), blood infections (acquired immunodeficiency syndrome) and hemorrhagic fever (yellow fever, Ebola hemorrhagic fever). Viruses are the most abundant and diverse biological entities on Earth and this is the reason for the high incidence of viral infections [2]. In addition, some viruses are etiological agents in the development of human tumors, particularly cervical cancer and hepatic cancer [3]. 

The main method and most cost-effective strategy for preventing viral infections is through vaccination, which is meant to prevent outbreaks by increasing immunity [4]. Vaccines for the prevention of several common acute viral infections, such as polio, rubella, measles, mumps, Influenza, yellow fever, encephalitis, rabies, smallpox and hepatitis B were developed during the 20th century and are available on a large scale [1,4]. Efforts to develop safe and effective vaccines against viruses that cause chronic infections, such as human immunodeficiency virus or hepatitis C virus did not give the expected results [1,4,5].

For many viral infections, only symptomatic treatment is indicated, while it is expected the immune system to fight off the virus. However, there are high-virulence viruses that cause serious viral infections where antiviral treatment is essential for patient survival. Although great efforts have been made to find effective medication, there are still no drugs that truly cure viral infections. Moreover, due to the ability of viruses to undergo rapid mutations, the mechanisms involved in developing resistance to antiviral drugs are activated in most cases [1,3]. As resistance toward antiviral drugs is becoming a global health threat, there is an intrinsic need to identify new scaffolds that are useful in discovering innovative, less toxic and highly active antiviral agents [3,6,7].

## 2. Nitrogen-Containing Heterocycles and Thiadiazole Ring in Biology and Medicinal Chemistry

Nitrogen-containing heterocycles are widely distributed in nature and are essential in vegetal and animal metabolism. They are found in nucleic acids, vitamins, antibiotics, alkaloids, etc. [8,9,10,11,12,13,14]. In addition, nitrogen-containing heterocycles are important targets for medicinal chemistry, as they are found in more than half of the commercially available drugs and can also act as versatile intermediates in the synthesis of complex products that exhibit outstanding biological activities [15]. Most of the nitrogen-containing heterocyclic compounds exhibit better biological activity than non-nitrogen compounds [12,16,17]. Currently, there are approximately 90 drugs approved for use in the treatment of nine human viral infections caused by human immunodeficiency virus (HIV), hepatitis B virus (HBV), hepatitis C virus (HCV), herpes simplex virus (HSV), Influenza virus, human cytomegalovirus (HCMV), varicella-zoster virus (VZV), respiratory syncytial virus (RSV) and human papillomavirus [18]. Most of these drugs (e.g., acyclovir, cidofovir, idoxuridine, nevirapine, pleconaril, ribavirin, etc.) are nitrogen heterocycle molecules [3,19].

Five-membered aromatic systems with three heteroatoms at symmetrical positions, such as the 1,3,4-thiadiazole ring, have been extensively studied due to their pharmacological profile and physicochemical and pharmacokinetic properties. 1,3,4-Thiadiazole derivatives are known as compounds having significant and diverse biological activities such as antibacterial, antifungal, antitubercular [20], analgesic and anti-inflammatory [21,22], antidepressant and anxiolytic [23], kinesin inhibitors [24], etc. The 1,3,4-thiadiazole ring is also found in several medicines such as acetazolamide, methazolamide, cefazolin, cefazedone, sulfamethizole or megazol [20,25,26,27]. The 1,3,4-thiadiazole nucleus provides the compounds with high lipophilicity and the ability to form mesoionic systems associated with discrete regions of positive and negative charges. These distinct features allow mesoionic compounds to efficiently cross cellular membranes, leading to good oral absorption, bioavailability and strong unique interactions with biological molecules (e.g., DNA, proteins, etc.) thus increasing the potential of 1,3,4-thiadiazole derivatives to exhibit biological activities [20,27,28,29]. The thiadiazole ring is a bioisostere of the pyrimidine and pyridazine rings. The pyrimidine nucleus is commonly found in naturally occurring compounds of biochemical importance, as well as in drugs (e.g., pyrimidine nucleosides and nucleotides, nucleic acids, antiviral drugs), while the pyridazine nucleus is a component of some pharmacologically active compounds [20,30,31,32]. The thiadiazole ring also acts as a bioisostere of the thiazole ring, and therefore thiadiazole derivatives can act in the same way as third- and fourth-generation antibacterial cephalosporins. This is an additional feature that highlights the high potential of this ring system in medicinal chemistry [20,28]. Many researchers suppose there is a connection between the biological potential of 1,3,4-thiadiazoles and the strongly aromatic character of the 1,3,4-thiadiazole ring, as well as the presence of the toxophoric =N-C-S- linkage group. Furthermore, the high in vivo stability and low toxicity for higher organisms are also attributed to the 1,3,4-thiadiazole ring [20,33,34]. In addition, the amine derivatives of 1,3,4-thiadiazole are also studied. 2-Amino-1,3,4-thiadiazole moiety and its derivatives are known for their antitumor, antitrypanosomal and uricogenic properties [20,25,27,35,36,37,38]. 2-Amino-1,3,4-thiadiazole derivatives are currently being synthesized in many laboratories, and in previous papers, we described several 2-amino-1,3,4-thiadiazoles that exhibit antibacterial, antifungal, antitubercular [20,39,40,41,42,43,44], and antiparasitic activities [27,38]. The purpose of this paper is to present some small molecules possessing the 2-amino-1,3,4-thiadiazole moiety that have shown antiviral activity.

## 3. The Activity of 2-Amino-1,3,4-Thiadiazole Derivatives Against Human Viral Pathogens

### 3.1. Human Immunodeficiency Virus (HIV)

About 37 million people infected with human immunodeficiency virus (HIV) (*Retroviridae* family) were reported in 2016 [45], with the highest incidence of infection in sub-Saharan Africa. In the Third World, HIV infection coupled with tropical diseases, malaria and tuberculosis causes a high level of mortality. Due to sexual transmission, acquired immunodeficiency syndrome (AIDS) affects many young workers and therefore the disease has not only a social impact, but also a significant economic impact in these regions [3].

In the past two and half decades, different organic compounds have been developed as drug candidates for the treatment of AIDS targeting one or more stages of the virus life cycle such as absorption, fusion, entry, un-coating, reverse transcription, integration, transcription and maturation [46]. HIV-1 reverse transcriptase (RT) is an essential enzyme that converts single-stranded RNA from the viral genome into double-stranded DNA before its integration into host DNA [47]. Since RT is a key enzyme in the life cycle of HIV-1, some HIV-1 RT inhibitors with nucleoside or non-nucleoside structures are currently used in AIDS treatment [48]. Nucleoside reverse transcriptase inhibitors (NRTIs) such as zidovudine, didanosine, zalcitabine, stavudine, lamivudine, abacavir, tenofovir or emtricitabine interact competitively with the catalytic site of the RT, while the non-nucleoside reverse transcriptase inhibitors (NNRTIs)—nevirapine, delavirdine, efavirenz, etc.—follow an allosteric interaction with a site adjacent to the NRTI binding site, the non-nucleoside inhibitor binding site (NNBS) [47]. Due to their high selectivity and low cytotoxicity, NNRTIs have gained an increasingly important role in HIV infection therapy [49]. Five drugs in the class of NNRTIs have been approved for the treatment of HIV infection: nevirapine, delavirdine and efavirenz as the first generation drugs and etravirine (Intelence tablets, Janssen Therapeutics Company, 2008) and rilpivirine (Edurant tablets, Janssen Therapeutics Company, 2011) as the next-generation NNRTIs [46,50,51,52]. Doravirine (MK-1439A) is a new NNRTI developed by Merck Company that completed two 48-week studies in 2017 [53]. Doravirine demonstrated antiretroviral activity and immunological effects similar to efavirenz with significantly fewer central nervous system adverse events [54]. In January 2018, US Food and Drug Administration (FDA) accepted for review two new drug applications (NDAs) for doravirine, as a once-daily tablet in combination with other antiretroviral agents and as a once-daily fixed-dose combination single tablet of doravirine with lamivudine and tenofovir disoproxil fumarate [55]. On 30 August 2018, FDA approved both applications of doravirine for AIDS treatment as Pifeltro tablets (doravirine 100 mg) and Delstrigo tablets (doravirine 100 mg, lamivudine 300 mg and tenofovir disoproxil fumarate 300 mg) [56,57]. Several NNRTIs (e.g., fosdevirine, lersivirine) underwent clinical development programs but were discontinued due to unfavorable pharmacokinetic, efficacy and/or safety factors [58,59,60].

Approximately 17 million patients have access to antiretroviral therapy capable of controlling viremia and reducing mortality [61]. However, long-term treatment with antiretroviral agents can lead to drug resistance due to rapid mutations in the viral genome resulting in RT mutations and HIV chemotherapy failure [62]. These concerns have attracted a particular focus on research into new antiretroviral drugs that address the limitations of currently available agents for the treatment of HIV infection [46,59].

As a bioisostere of pyrimidine, a nucleoside component of nucleic acids, the thiadiazole ring can impart antiviral activity [28]. Studies concerning the antiviral activity of 1,3,4-thiadiazole derivatives often gave compounds with moderate or lower in vitro anti-HIV-1 and anti-HIV-2 activity than the reference drugs [63,64]. A relevant example is the chiral 2-substituted 5-(4-chlorophenylamino)-1,3,4-thiadiazoles **1**–**5** synthesized by Akhtar et al. by acidic cyclodehydration of the corresponding thiosemicarbazides [65].


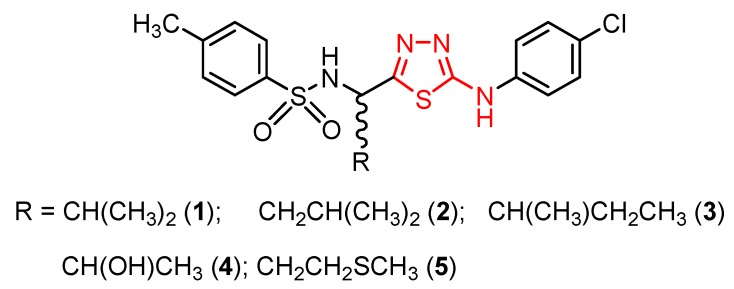


**Figure 1:**
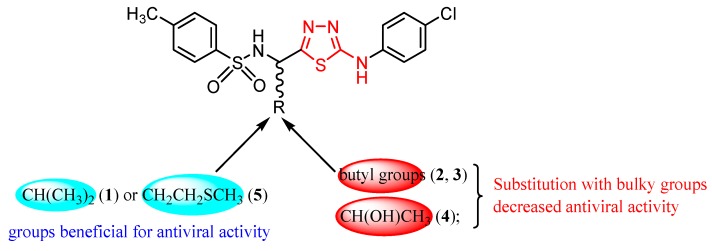
Influence of substituents of 1,3,4-thiadiazole derivatives **1**–**5** on anti-HIV activity.

In vitro HIV inhibitory activity using human T-lymphocyte (MT-4) cells gave moderate or low half-maximal effective concentration (EC_50_) values in comparison to efavirenz (EC_50_ value of 0.003 μg/mL). The best results were obtained for derivatives **1** and **5** (Figure 1) with moderate EC_50_ values. Thus, compound **1** showed EC_50_ > 14 μg/mL against HIV-1 (strain IIIB) and EC_50_ > 12.4 μg/mL against HIV-2 (strain ROD), while the compound **5** showed EC_50_ > 12.6 μg/mL against HIV-1 and EC_50_ > 12.5 μg/mL against HIV-2. Low values for cytotoxicity concentration 50% (compound concentration that reduces the viability of mock-infected MT-4 cells by 50%), namely the CC_50_ value of 14.0 ± 1.2 μg/mL for compound **1** and 13.3 ± 0.8 μg/mL for compound **5**, respectively, resulted in low selectivity index SI ≤ 1 (SI = CC_50_/EC_50_). Other derivatives showed lower activity with EC_50_ within the range of 47.4–125 μg/mL. However, chemical modifications on this scaffold might lead to compounds with enhanced activity as NNRTIs [65].

Some 1,3,4-thiadiazole derivatives obtained by Hamad et al. [66] from amino acid analogs were screened for anti-HIV-1 (strain IIIB) and anti-HIV-2 (strain ROD) activity by the inhibition of the virus-induced cytopathic effect in human MT-4 cells based on 3-(4,5-dimethylthiazol-2-yl) -2,5-diphenyl tetrazolium bromide (MTT) assay. 2-(Naphthalen-2-yloxy)-*N*-((5-(phenylamino) -1,3,4-thiadiazol-2-yl)methyl)acetamide **6** showed in vitro inhibitory activity with EC_50_ values of 0.96 μg/mL (HIV-1 strain IIIB) and 2.92 μg/mL (HIV-2 strain ROD), respectively, but low selectivity (SI < 1). Structure–activity relationship (SAR) studies have suggested that the substitution of the acetamide moiety with a thiadiazole ring may lead to more active derivatives compared to other compounds bearing different heterocyclic rings. Even though anti-HIV activity and selectivity of derivative **6** are limited compared to efavirenz (EC_50_ value of 0.003 μg/mL and SI ≈ 13333), it may serve as the basis for future modification in the search for new potent non-nucleoside antiviral agents [66].


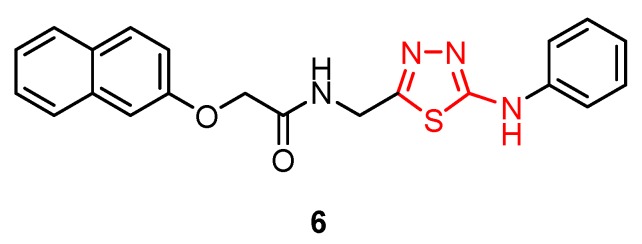


A new class of HIV-1 NNRTIs, *N*-aryl-2-arylthioacetamides, has been identified in the last years [67,68,69,70]. Studies on the crystalline structure of the RT-NNRTI complex suggested that NNRTIs have a common mode of action and interact with a hydrophobic pocket. *N*-aryl-2-arylthioacetamides adopted a butterfly-like conformation in which the arylthio moiety and the phenyl ring mimic the butterfly wings. SAR studies showed that the arylthio moiety strongly influenced the antiviral activity, leading to different results depending on the steric/electronic properties of the groups [47]. Based on these findings, Xiaohe et al. synthesized 2-(5-amino-1,3,4-thiadiazol-2-ylthio)-*N*-(aryl) acetamide derivatives **7**–**10** as new NNRTIs [47].


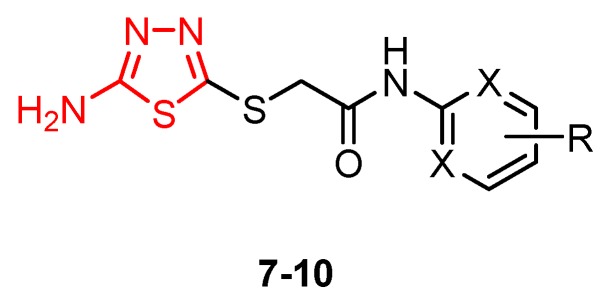


**Figure 2:**
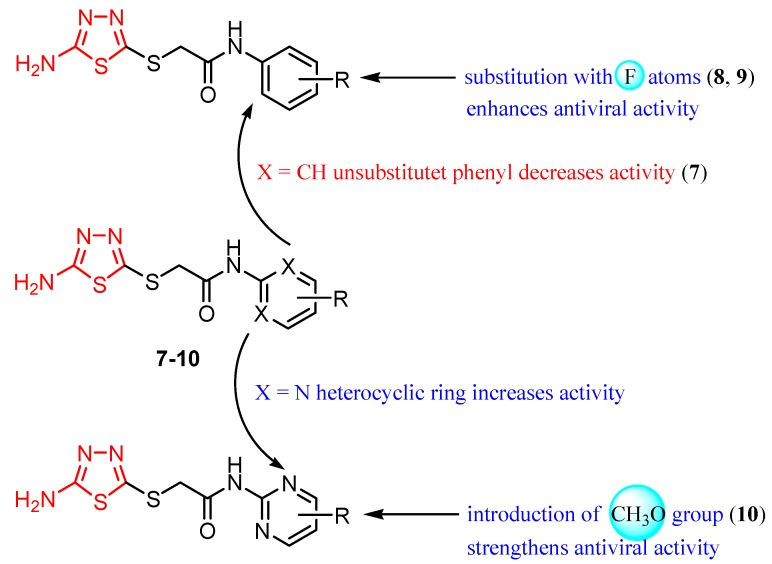
The influence of substituents of 1,3,4-thiadiazole derivatives **7**–**10** on anti-HIV activity.

Although they exhibited less anti-HIV-1 activity compared to standard drug zidovudine (half-maximal inhibitory concentration IC_50_ = 0.016 μM), these compounds showed significant anti-HIV-1 activity at micromolar concentrations (IC_50_ within the range of 7.50–20.83 μM) (Table 1). It has been observed that the 2-amino-1,3,4-thiadiazole moiety may be a good group for anti-HIV-1 activity by providing promising antiviral agents. Moreover, the electronic properties of the *N*-aryl group influenced antiviral potency. The introduction of electron-withdrawing groups, such as fluorine or trifluoromethyl on phenyl ring (derivatives **8** and **9**), enhanced antiviral activity compared to the unsubstituted phenyl derivative **7**. SAR studies suggested that the steric/electronic properties of the *N*-phenyl substituents influenced the antiretroviral activity more than their positions. In addition, the nature of the *N*-aryl ring influenced the antiviral potency as can be observed for derivative **10** with a pyrimidyl ring which was the most active compound (Figure 2) [47].

Molecular modeling studies for the pyrimidyl derivative **10** showed the formation of two potential intermolecular hydrogen bonds involving a nitrogen atom and the amino group of the thiadiazole ring and aminoacids from NNBS of RT. In addition, the electron-deficient pyrimidine ring of the ligand establishes π–π interactions with the electron-rich benzene rings of RT. Despite the docking simulation results, the inhibitory activity of compound **10** against HIV-1 (strain IIIB) replication in MT-4 cell culture was lower than that of zidovudine. However, given the butterfly-like orientation as a necessary structural condition for antiretroviral activity, these results reveal the promising inhibitory potential of this scaffold [47].

The series of 5-(pyridin-2-ylmethyl)-1,3,4-thiadiazol-2-amine derivatives **11**–**16** were synthesized by intramolecular cyclization of the corresponding hydrazinecarbothioamides under acidic conditions and the activity against HIV-1 was tested on MT-4 cells by MTT assay using efavirenz as a standard drug [64].


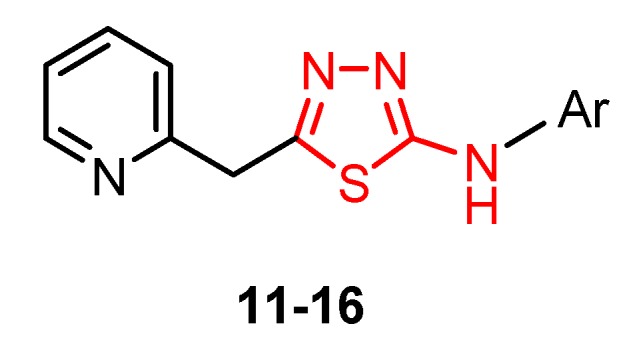


**Figure 3:**
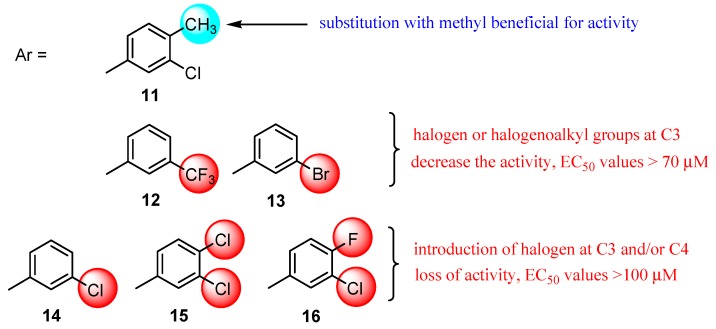
The influence of substituents of 1,3,4-thiadiazole derivatives **11**–**16** on anti-HIV activity.

Except for derivative **11** which showed low activity (EC_50_ values of 47 μM), the activity of the other compounds was even lower (EC_50_ values > 70 μM) proving that substitution with halogens or halogenoalkyl groups at C3 and/or C4 of phenyl ring led to a decrease or loss of activity (Figure 3). Although pyridine derivatives did not exhibit selective anti-HIV-1 activity, the fact that compound **11** showed some antiviral activity may encourage further research on this structure. Subsequent chemical modifications by substitutions on aromatic rings with different groups may lead to compounds with improved activity [64].

The invasion of the central nervous system (CNS) by the HIV-1 virus frequently causes brain inflammation and progressive neurological diseases, which are commonly referred to as HIV associated neurocognitive disorders (HAND) [71]. HAND affects 7%–15% of AIDS patients and is characterized by neuronal dysfunction including synaptic damage, neuronal degeneration and cell dropout [72]. Cells involved in HAND pathogenesis are macrophages and microglia, which are the main targets of HIV-1 infection in the brain. When infected with HIV-1, macrophages and microglia increase the production and release of several soluble neurotoxic factors, such as glutamate, inducing neuronal damage [71]. 

Glutamine is the most abundant amino acid in the human body and is involved in more metabolic processes than any other amino acid. Glutamine is produced from glutamate and ammonia by the enzyme glutamine synthetase [73,74]. The conversion of glutamine to glutamate is catalyzed by the mitochondrial enzyme, glutaminase [72,75]. The glutamine/glutamate cycle in the human body plays several important metabolic functions. Thus, glutamine and glutamate are precursors to the biosynthesis of proteins, neurotransmitters, nucleotides, nucleic acids and other important biological molecules. The glutamine/glutamate cycle is the substrate for the synthesis of urea in the liver, genesis of ammonia in the kidneys and for hepatic and renal gluconeogenesis. The glutamine/glutamate exchange regulates the acid-base balance in kidneys, acts as an oxidative fuel for the intestines and cells of the immune system and provides the transport of nitrogen between organs [74,75,76]. The existence of a glutamine/glutamate cycle in CNS was confirmed in the last years [76,77]. Phosphate-activated mitochondrial glutaminase is the predominant enzyme that uses glutamine in the brain. Glutamine is present in the extracellular fluid of the brain at high concentrations and provides an abundant substrate for glutaminase [72,75]. Therefore, it has been hypothesized that mitochondrial glutaminase activation is responsible for the high levels of glutamate in the brains of HIV-1 infected patients [72]. While glutamate mediates different physiologic processes, elevated extracellular concentrations of glutamate can induce neuronal damage (e.g., dementia, brain atrophy) [71,72].

**Figure 4:**
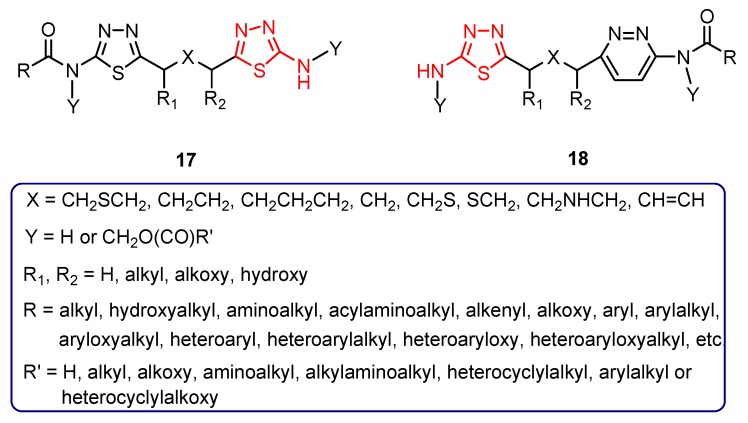
The general formula of glutaminase inhibitors **17** and **18.**

Some glutaminase inhibitors (e.g., 6-diazo-5-oxo-*L*-norleucine, etc.) were studied in vitro for their ability to prevent the generation of glutamate by HIV-1 infected macrophages. The results support the hypothesis that glutaminase mediates glutamate generation in HIV-infected human macrophages. When glutaminase was inhibited by various inhibitors, HIV-induced glutamate production decreased and the neuronal damage was diminished [71,72,78]. Furthermore, for some glutaminase inhibitors, a non-competitive mechanism of inhibition has been described [78]. These findings support glutaminase as a potential component of the HAND process and can provide a new therapeutic target for the treatment of neurocognitive disorders associated with HIV infection [71,72,78]. In connection with these results, a large number of glutaminase inhibitors having a bis-thiadiazole (**17**) and a pyridazine-thiadiazole (**18**) skeleton (Figure 4), respectively, were synthesized as a method of treating or preventing multiple viral infections, including infections with retroviruses [75].

Multiple experiments were performed in order to study the biological profile of the compounds. Some of the synthesized derivatives are prodrugs, which under physiologic conditions (in vivo), are converted into the therapeutically active parent compound. Studies have also been conducted to obtain pharmaceutical preparations suitable for use in human patients comprising any of the synthesized derivatives and one or more pharmaceutically acceptable excipients. In addition, the authors assume that the derivatives may be used alone or in combination with known antiviral drugs. Studies on kidney-type glutaminase inhibition showed good results for many derivatives such as compound **19** with an IC_50_ value of 0.24 μM and its deuterium derivative **20** with an IC_50_ value of 0.54 μM [75].


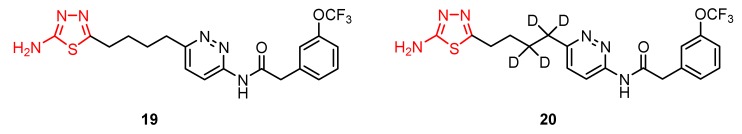


### 3.2. Human Cytomegalovirus (HCMV)

Human cytomegalovirus (HCMV, *Herpesviridae* family) is a ubiquitous deoxyribonucleic acid virus that infects people of all ages [79,80]. HCMV infection can be acquired through horizontal and vertical transmission. HCMV spreads from infected people through direct contact with body fluids that carry the virus, such as urine, saliva, cervicovaginal secretions, sperm and breast milk. Vertical transmission through organ transplantation, from mother to child or transmission via blood transfusion, is also possible [79,80]. Blood tests indicate that 60%–90% of the adult population experienced HCMV infection at some time during their life [81]. Although most of these infections are asymptomatic, certain patient groups such as babies that are infected before birth and children or adults with weakened immune systems due to diseases or medications (e.g., HIV-infected patients, organ transplant recipients) can develop severe illnesses that require medical treatment [79]. HCMV is able to remain latent in several cells of the human body for a long time and can be reactivated if the person develops immune system suppression [79,82].

The first-line drugs recommended for the treatment of HCMV infection are intravenous ganciclovir or orally administered valganciclovir [83]. Although tolerability of ganciclovir and valganciclovir is acceptable, hematological or neurological side effects can occur. Neutropenia, thrombocytopenia and anemia are the main toxic effects that limit therapy with these drugs. Serum creatinine levels may increase during ganciclovir therapy, which requires monitoring of renal function [84]. Encephalopathy is the neurotoxic effect of ganciclovir and valganciclovir [85]. Foscarnet is also a very effective anti-HCMV drug, and cidofovir is a broad-spectrum antiviral with good activity against HCMV. Both drugs cause a high level of nephrotoxicity that limits treatment [83].

Novel 2-amino-1,3,4-thiadiazole derivatives with antiviral activity against HCMV have been patented [86]. A large number of 472 synthesized compounds were tested in an HCMV polymerase assay at a concentration of 25 μM. The degree of enzyme inhibition ranged from 20% to 100%. Among the most active compounds, four derivatives exhibited a 100% inhibition rate, 29 derivatives showed an inhibition rate of 90.1%–99.9% and 16 derivatives showed an inhibition rate of 80.3%–89.9%. Three structural series stand out among the most active 1,3,4-thiadiazole derivatives: 1,3-dioxo-1,3-dihydro-2-benzofuran-5-carboxamide derivatives such as **21**–**30**, 9-octadecenamide derivatives such as **31**–**38** and 2-ethoxy-1-naphthamide derivatives such as **39**–**42** (Table 2, Table 3 and Table 4). Most of the derivatives belong to the 1,3-dioxo-1,3-dihydro-2-benzofuran-5-carboxamide series. At the same time, the most active compounds belong to this series, so it can be concluded that the 1,3-dioxo-1,3-dihydro-2-benzofuran-5-carboxamide moiety is a good scaffold for anti-HCMV activity [86].


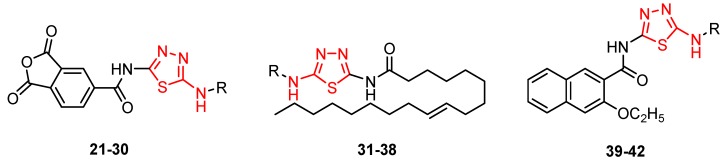


Other compounds also showed good inhibitory activity. These derivatives contain a five- to six-membered saturated heterocyclic moiety, such as imidazolidinyl, tetrahydrofuryl, piperidinyl, morpholinyl, thiomorpholinyl or 5- to 10-membered aromatic or unsaturated heterocyclic moiety such as furyl, pyrrolyl, pyridyl, benzothiazolyl, etc. (e.g., derivatives **43**,**44**) [86].


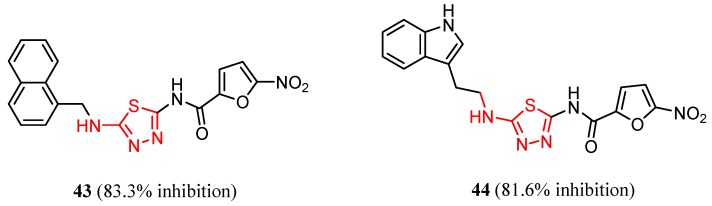


While the synthesized derivatives have shown inhibitory activity against HCMV polymerase, their antiviral activity cannot be limited to a specific mechanism of action. These compounds may be active against cytomegalovirus by HCMV polymerase inhibition or by other mechanisms of action. In addition, during the experiments, many of these compounds also showed activity against other herpes viruses, such as varicella-zoster virus (VZV), Epstein–Barr virus (EBV), herpes simplex virus (HSV), and human herpesvirus type 8 (HHV-8). Pharmaceutical compositions containing such compounds or their pharmaceutically acceptable salts useful as antiviral agents have also been studied. Studies have been conducted for the administration of pharmaceutical preparations by parenteral, topical, oral or rectal route, depending on the purpose of their use to treat internal or external viral infections [86].

### 3.3. Respiratory Viruses

Acute respiratory infections are a major global health problem responsible for about 3.9 million deaths worldwide each year [87,88]. These infections are of the top five causes of mortality worldwide and the leading cause of mortality among children under five years of age in many developing countries [87,89]. Acute respiratory infections are most often caused by viruses. Over 200 viral serotypes are associated with human respiratory diseases [90] including Influenza A and Influenza B virus, respiratory syncytial virus (RSV), parainfluenza virus (PIV), human adenovirus (HAdV), human coronavirus (HCoV), human rhinovirus (HRV), human metapneumovirus (HMPV) and human bocavirus (HBoV). In addition, two human polyomaviruses (HPyV), KIPyV and WUPyV, have been detected in patients with respiratory infections [91]. These infections affect all age groups, but nearly all severe episodes occur in children under five years, the elderly and immunocompromised individuals (e.g., HIV-infected patients) [87,89]. In adults, viral respiratory infections are the cause of 30%–50% of pneumonia cases, 80% of asthma complications and 20%–60% of chronic obstructive pulmonary disease exacerbations [87]. Consequently, common viral respiratory infections cause a greater economic burden than many other clinical conditions in terms of medical expenses and productivity losses [87,92]. The World Health Organization has supported the monitoring of acute respiratory diseases worldwide since 1977 [91].

The Influenza virus belongs to the *Orthomyxoviridae* family and causes respiratory infections in about 20% of the global population every year. The 1918 flu pandemic was caused by Influenza A subtype H1N1 and killed 50 million people around the world [93]. The Asian Influenza caused by Influenza A subtype H2N2 occurred in 1957 and the Hong Kong Influenza caused by Influenza A subtype H3N2 took place in 1968 and made far fewer victims than the 1918 Spanish flu. About 70 people died in Asia in 2004–2005 due to the H5N1 strain of avian flu [93]. The 2009 flu pandemic (swine flu) was the second pandemic involving a strain of Influenza A virus. It was classified as Influenza A H1N1 2009 and the genetic material originated from three different species: human, avian and swine [7,94]. The chemotherapy or prophylaxis of Influenza infections comprises agents blocking the Influenza A virus M2 proton-selective ion channel (amantadine, rimantadine) and neuraminidase inhibitors (zanamivir, oseltamivir, laninamivir, peramivir) [1,93]. Both classes can induce virus resistance and therefore there is an urgent need to develop new antiviral agents with novel mechanisms of action. An alternative concept has recently emerged and it is based on the idea of designing new molecules targeting host cell factors that are hijacked by the virus during its replication. Host-targeting antivirals are an alternative strategy for addressing host structures involved in the virus life cycle. This type of inhibitors could exhibit a significantly greater barrier for selecting drug-resistant viruses and, in addition, display broad-spectrum antiviral activity when interacting with a cellular target common to several viruses. The host factor-directed antiviral therapy is recently studied. This is increasingly recognized as a relevant approach to combat viral resistance and provides broad-spectrum antiviral agents [95,96].

Many studies are currently being developed to find new Influenza inhibitors. Tatar et al. synthesized 2-phenylamino-1,3,4-thiadiazole derivatives **45**–**48 [49]**.


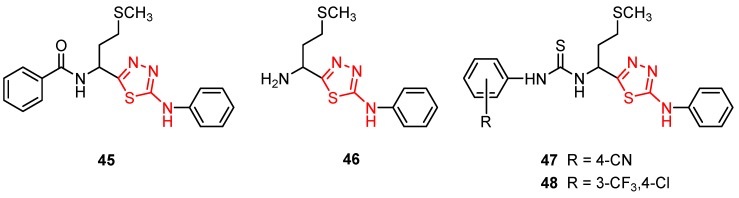


The antiviral activity against some respiratory viruses such as Influenza A H1N1, Influenza A H3N2, Influenza B, Parainfluenza-3, RSV, Reovirus-1and Feline Coronavirus was investigated and the results are summarized in Table 5. No activity was observed at the highest concentration tested or at subtoxic concentration against Influenza B and RSV [49].

**Figure 5:**
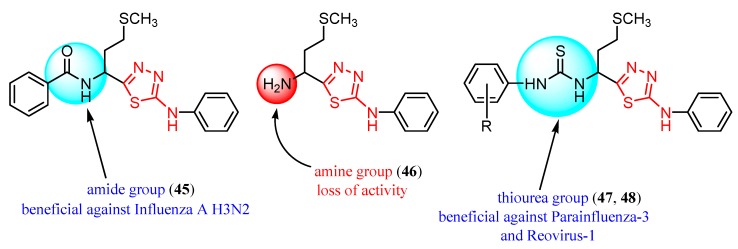
The influence of substituents of 1,3,4-thiadiazole derivatives **45**–**48** on anti-respiratory viruses’ activity.

The in vitro antiviral assay showed *N*-{3-(methylthio)-1-[5-(phenylamino)-1,3,4-thiadiazol-2-yl]propyl}benzamide **45** as an Influenza A H_3_N_2_ virus subtype inhibitor. With an EC_50_ value of 31.4 μM, the derivative **45** was the most potent among the tested compounds and moderate active compared to standard drug oseltamivir, but a promising scaffold for future developments. Derivatives **47** and **48** exhibited activity against Parainfluenza-3 and Reovirus-1 and probably the thiourea moiety favors antiviral activity on these strains (Figure 5) [49].

### 3.4. Hepatitis Viruses

Viral hepatitis is a liver inflammation responsible for about 171,000 deaths every year in the European Region. Patients may have an acute form as a recent infection, with relatively rapid onset or a chronic form. There are five main hepatitis viruses (HAV, HBV, HCV, HDV and HEV) with different ways of transmission and different impact on human health [97]. While HAV or HEV infection is usually mild, with most people recovering quickly and completely, infection with HBV, HCV or HDV often leads to chronic infections and progressive liver damage with the development of cirrhosis and liver cancer [97]. There are about 15 million people living with chronic HBV infection and about 14 million with HCV infection in the European Region [97]. Safe and effective vaccines for the prevention of HBV infection have been available since the 1990s. These vaccines also provide protection from HDV infection. Unfortunately, the HCV vaccine has not yet been developed [97,98]. Many patients infected with HBV are adults born before the hepatitis B vaccine became available in the 1990s. In these cases, drug treatment is the only option [98]. Several nucleoside and non-nucleoside derivatives with anti-HBV (e.g., adefovir, entecavir, lamivudine, telbivudine, tenofovir) or anti-HCV activity (e.g., boceprevir, grazoprevir, elbasvir, ledipasvir, sofosbuvir, telaprevir) are in use [3,99] and chronic infections with HBV and HCV can be currently controlled or even cured. Due to the costs of antiviral drugs for chronic hepatitis, access to treatment is a major obstacle in many countries and finding new, less expensive antiviral drugs is a necessity [97].

1,2-Dihydro-4,6-dimethyl-2-oxo-1-[(5-(phenylamino)-1,3,4-thiadiazol-2-yl)methyl]pyridine-3-carbonitrile **49** was prepared from the corresponding phehylthiosemicarbazide by cyclization in sulfuric acid [6]. The derivative **49** was tested for antiviral activity against the hepatitis B virus (HBV) using HepG2.2.2.15 cell line, a human hepatoblastoma cell line that produces HBV viral particles. Cytotoxicity was also tested by the cell viability method (MTT assay). Preliminary screening indicated high inhibitory activity against HBV with an IC_50_ value of 0.3 μM, low cytotoxicity (CC_50_ value of 333.3 μM) and a selectivity index SI of 1111 compared to the standard drug lamivudine (IC_50_ value of 0.1μM). From these results, it can be concluded that pyridine-2-one may be a good substituent on the 1,3,4-thiadiazole ring, and these two moieties together make a promising scaffold in promoting anti-HBV activity [6].


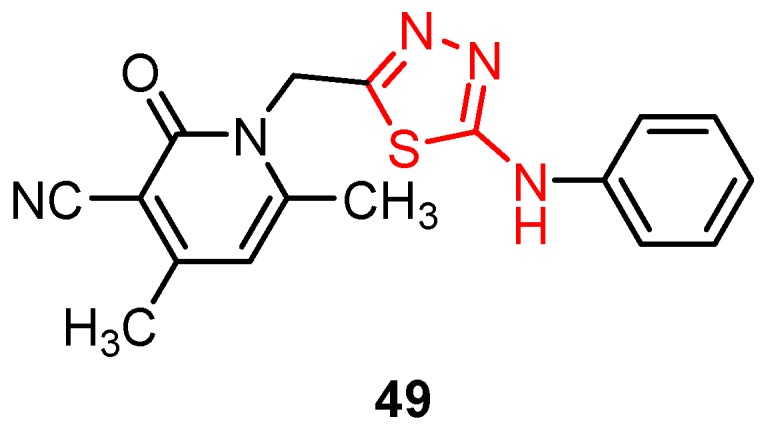


### 3.5. Miscellaneous Viruses

Sindbis fever, a less common human viral disease, is caused by a mosquito-borne virus called Sindbis virus (*Togaviridae* family). Despite the wide distribution of Sindbis virus, symptomatic infections in humans have been reported in only a few limited geographical areas such as northern Europe (Finland, Sweden and Russia), South Africa, Australia and China [100]. 1,3,4-Thiadiazole derivatives **50**–**55** were tested for antiviral activity against several viruses [101].


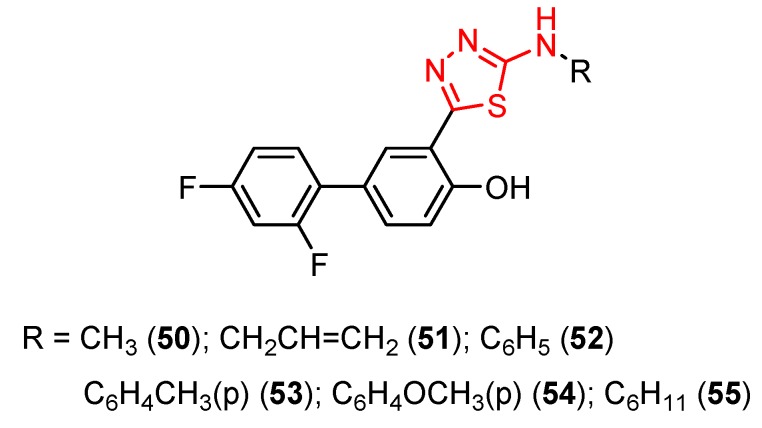


**Figure 6:**
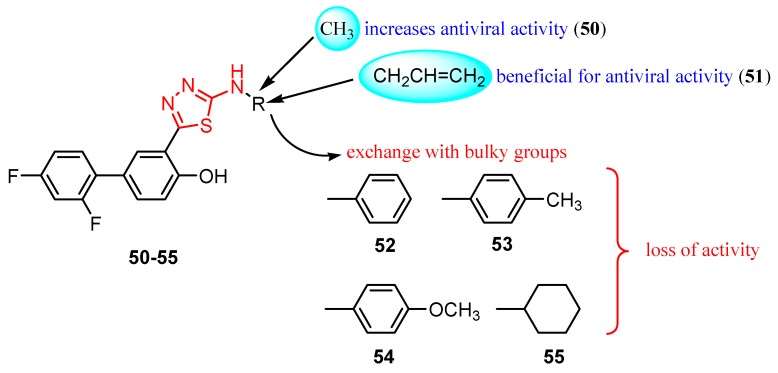
The influence of substituents of 1,3,4-thiadiazole derivatives **50**–**55** on viral inhibition.

The derivatives **50** (methyl) and **51** (allyl) showed antiviral activity against herpes simplex virus-1 TK-KOS and herpes simplex virus-1 TK-KOS ACV, Sindbis virus, Coxsackie virus B4 and Punto Toro virus at a concentration of 16 μg/mL. The highest antiviral activity was exhibited against Sindbis virus by derivative **50** at a concentration of 9.6 μg/mL. It seems that the size of the amino group substituent influenced the antiviral activity. While derivatives **50** and **51** with small alkyl groups showed antiviral activity, compounds with bulky aromatic (**52**, **53** and **54**) or cycloalkyl (**55**) groups were not capable of viral inhibition (Figure 6) [101].

**Figure 7:**
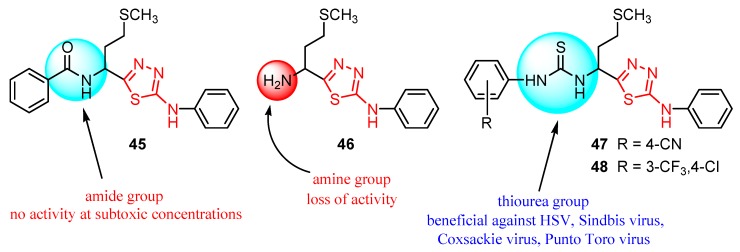
The influence of substituents of 1,3,4-thiadiazole derivatives **45**–**48** on antiviral activity.

2-Phenylamino-1,3,4-thiadiazole derivatives **45**–**48** were also screened against herpes simplex HSV-1 and HSV-2, herpes simplex virus-1 TK-KOS ACV, Sindbis virus, Coxsackie virus B4 and Punto Toro virus (Table 6) [49]. While amide **45** and amine **46** did not exhibit antiviral activity at subtoxic concentrations, *in vitro* tests showed antiviral activity for thiourea derivatives **47** and **48**, highlighting what other studies have reported that derivatives bearing the -NH-CS-NH- group have demonstrated antiviral activity. Derivative **48** exhibited activity against different strains of HSV and both derivatives **47** and **48** showed activity against Sindbis virus, Coxsackie virus B4 and Punto Toro virus (Figure 7) [49].

2-Amino-5-(2-sulphamoylphenyl)-1,3,4-thiadiazole **56** reduced the replication of some DNA viruses such as adenovirus Ad17 and herpes simplex HSV-1 and RNA viruses such as Poliovirus 1, Echovirus 2 and Coxsackie virus B4 at concentrations ranging from 20 to 100 μg/mL [102,103]. In vitro experiments were performed using samples of 10^6^ human aneuploid HEp-2 cells that were infected with 10 infectious units per cell. Derivative **56** was highly active against all viral strains, significantly reducing viral replication at a concentration of 50 μg/mL. The best inhibition was recorded against Echovirus 2 virions that were completely inhibited at a concentration of 20 μg/mL (Table 7). Regarding the mechanism of action, the authors assume that compound **56** may act on the viral structural proteins preventing the assembly of virus particles [102].

**Figure 8:**
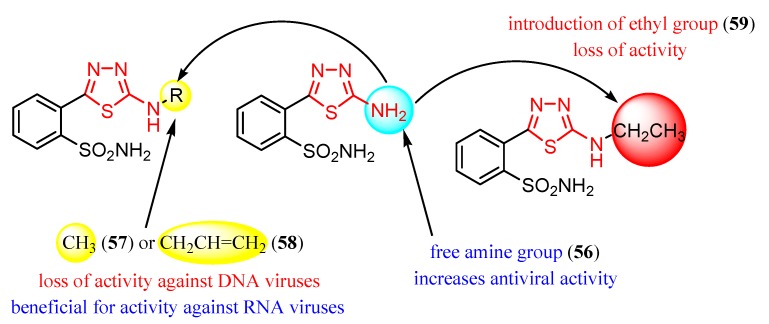
The influence of substituents of 1,3,4-thiadiazole derivatives **56**–**59** on antiviral activity.

Derivatives of compound **56** were prepared. Methyl derivative **57** and allyl derivative **58** reduced the replication of RNA viruses (Poliovirus 1 and Coxsackie virus B4) at concentrations of 50 and 100 μg/mL, while ethyl derivative **59** was completely inactive against all viral strains (Table 7). These results suggest the importance of the side chain for antiviral activity (Figure 8) [102].


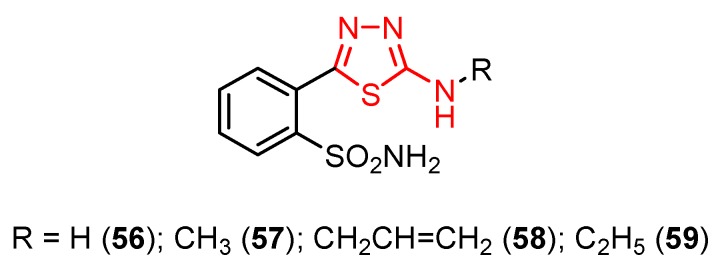


**Figure 9:**
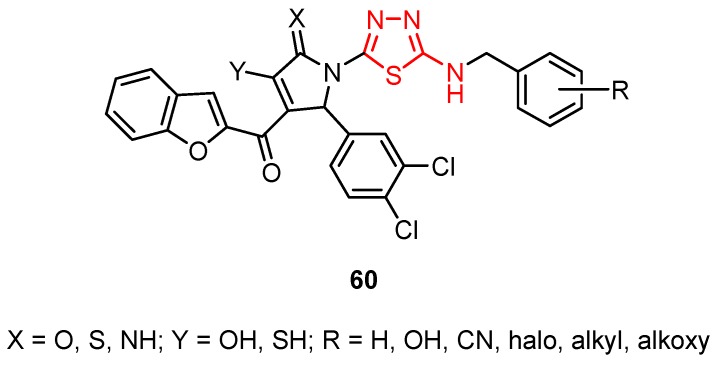
The general formula of derivatives **60.**

Cui et al. synthesized several pyrrolyl-1,3,4-thiadiazoles with general formula **60** (Figure 9). The compounds showed antiviral activity against some viruses of the *Flaviviridae* family such as West Nile virus and dengue virus [25,104].

The use of non-nucleoside derivatives as antiviral chemotherapeutic agents has stimulated extensive research into the synthesis of compounds of this class. However, many antiviral drugs are nucleoside analogs that act by suppressing the synthesis of viral DNA or RNA which leads to inhibition of virus replication or cell division. Research has been carried out to find new nucleoside antiviral agents in which the natural nucleobases have been replaced by heterocyclic rings, as can be seen in derivatives **61**–**64** [105].


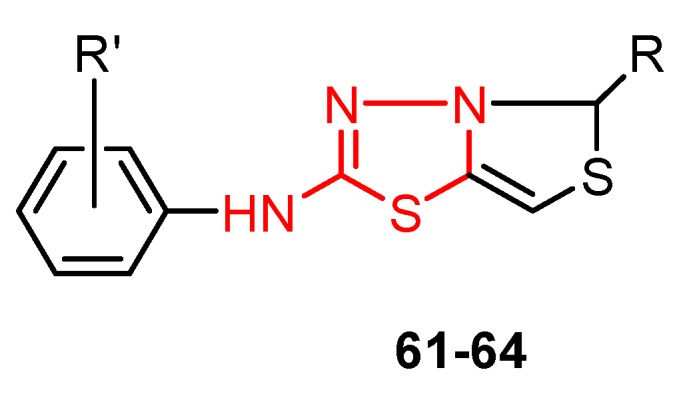


**Figure 10:**
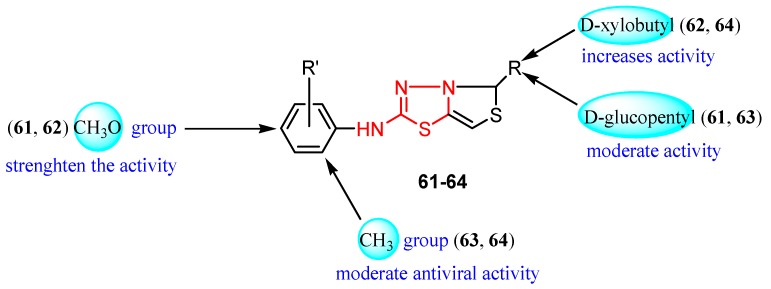
Influence of substituents of 1,3,4-thiadiazole derivatives **61**–**64** on antiviral activity.

The antiviral activity was evaluated *in vitro* against viral strains parasitizing *Chenopodium amaranticolor*. The ability of derivatives **61**–**64** to control the viral infection of *Chenopodium amaranticolor* leaves was studied at two concentrations: 1000 ppm and 100 ppm. Generally, the compounds showed good rates of viral infection control at 1000 ppm. The best results were observed for the derivatives bearing the D-xylobutyl group (compound **62**—82% control and derivative **64**—76% control). The substituent on the aryl ring did not significantly influence biological activity, although the compounds **61** and **62** having a methoxy group were slightly more active than derivatives **63** and **64** bearing a methyl group (Table 8, Figure 10). The study may be useful in obtaining new pesticides for agriculture [105].

## 4. Conclusions

The research focused on 1,3,4-thiadiazole derivatives indicates a broad spectrum of pharmacological activities associated with good physicochemical and pharmacokinetic properties. This article presents a literature review of 2-amino-1,3,4-thiadiazole derivatives that have been evaluated for antiviral activity against several viral strains. In addition to the 2-amino-1,3,4-thiadiazole moiety, antiviral activity is also dependent on the nature of the substituents, and structure–activity studies have shown the most efficient substituents for antiviral activity in each class. Based on the literature data, the 2-amino-1,3,4-thiadiazole scaffold may be considered a possible pharmacophore group that can be incorporated into the structure of known compounds to enhance antiviral activity and contributes to the search and development of new medicines as an alternative to the treatment of viral infections.

## Figures and Tables

**Table 1:** Structural details and IC_50_ values of compounds **7**–**10** (adapted from [47]).

	X	R	IC_50_ (μM)
**7**	CH	H	20.83 ± 1.17
**8**	CH	2,5-F_2_	16.10 ± 0.24
**9**	CH	3,5-(CF_3_)_2_	14.93 ± 0.84
**10**	N	4,6-(OCH_3_)_2_	7.50 ± 1.06

**Table 2:** Structural details and human cytomegalovirus (HCMV) polymerase inhibition values of compounds **21**–**30** (adapted from [86]).

	R	Inhibition (%)		R	Inhibition (%)
**21**	(CH_2_)_10_CH_3_	100	**26**	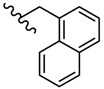	99.8
**22**	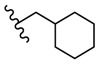	100	**27**	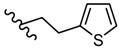	99.8
**23**	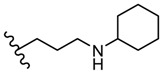	100	**28**	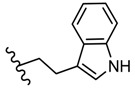	99.7
**24**	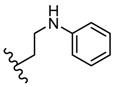	99.9	**29**	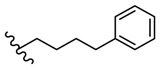	99.6
**25**	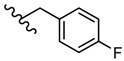	99.8	**30**	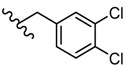	99.5

**Table 3:** Structural details and HCMV polymerase inhibition values of compounds **31**–**38** (adapted from [86]).

	R	Inhibition (%)		R	Inhibition (%)
**31**	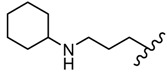	100	**35**	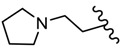	97.7
**32**	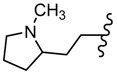	99.9	**36**	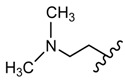	97.0
**33**	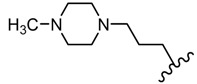	99	**37**	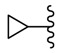	92.6
**34**	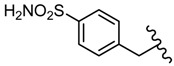	98.3	**38**	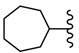	91.8

**Table 4:** Structural details and HCMV polymerase inhibition values of compounds **39**–**42** (adapted from [86]).

	R	Inhibition (%)		R	Inhibition (%)
**39**	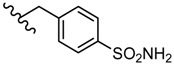	93.1	**41**	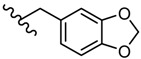	83.9
**40**	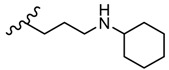	92.9	**42**	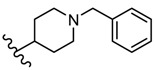	83.1

**Table 5:** Antiviral evaluation and in vitro cytotoxicity of compounds **45**–**48** (adapted from [49]).

	Influenza A H1N1		Influenza A H3N2		Para Influenza-3		Reovirus-1		Feline Coronavirus	
	EC_50_ (μM)	CC_50_ (μM)	EC_50_ (μM)	CC_50_ (μM)	EC_50_ (μM)	MCC (μM)	EC_50_ (μM)	MCC (μM)	EC_50_ (μM)	CC_50_ (μM)
**45**	42	> 100	31.4	> 100	> 20	≥ 20	> 20	≥ 20	> 100	> 100
**46**	-	23	-	23	> 100	>100	> 100	> 100	> 100	> 100
**47**	-	79	-	79	> 20	100	> 20	100	> 100	> 100
**48**	-	2.7	-	2.7	> 4	20	> 4	20	> 4	11
Oseltamivir carboxylate	4.7	> 100	9	> 100	-	-	-	-	-	**-**
Ribavirine	8	> 100	8.1	> 100	50	> 250	> 250	> 250	-	-
Amantadine	127	> 500	1.7	> 500	-	-	-	-	-	-

MCC: minimum cytotoxic concentration.

**Table 6:** Antiviral evaluation and in vitro cytotoxicity of compounds **45**–**48** (adapted from [49]).

	HSV-1		HSV-2		HSV-1 (TK-KOS ACV)		Sindbis Virus		Coxsackie Virus B4		Punto Toro Virus	
	EC_50_ (μM)	MCC (μM)	EC_50_ (μM)	MCC (μM)	EC_50_ (μM)	MCC (μM)	EC_50_ (μM)	MCC (μM)	EC_50_ (μM)	MCC (μM)	EC_50_ (μM)	MCC (μM)
**45**	> 20	≥ 20	> 20	≥ 20	> 20	≥ 20	> 20	≥ 20	> 20	≥ 20	> 20	≥ 20
**46**	> 100	> 100	> 100	> 100	> 100	> 100	> 100	> 100	> 100	> 100	> 100	> 100
**47**	> 100	> 100	> 100	> 100	> 100	> 100	> 20	100	> 20	100	> 20	100
**48**	> 20	100	> 20	100	> 20	100	> 4	20	> 4	20	> 4	20
Acyclovir	0.9	> 250	0.4	> 250	> 250	> 250	-	-	-	-	-	-
Ribavirine	-	-	-	-	-	-	> 250	> 250	> 250	> 250	112	> 250

MCC: minimum cytotoxic concentration.

**Table 7:** Antiviral evaluation and in vitro cytotoxicity of compounds **56**–**59** (adapted from [102]).

	Concn (μg/mL)	Ad17 (Cells Number)	HSV-1 (Cells Number)	Poliovirus 1 (Cells Number)	Echovirus 2 (Cells Number)	Coxsackie virus B4 (Cells Number)	MNC (μg/mL)
**blank**		5 × 10^8^	3 × 10^6^	3 × 10^9^	2 × 10^9^	3 × 10^8^	
**56**	20	7 × 10^6^	6 × 10^5^	4 × 10^6^	0	6 × 10^5^	900
	50	4 × 10^2^	5 × 10^4^	2 × 10^3^	0	2 × 10^3^	
	100	2 × 10^2^	3 × 10^4^	1 × 10^3^	0	2 × 10^3^	
**57**	50	6 × 10^8^	3 × 10^6^	2 × 10^5^	-	8 × 10^4^	800
	100	6 × 10^8^	2 × 10^6^	8 × 10^4^	-	2 × 10^4^	
**58**	50	4 × 10^8^	2 × 10^6^	3 × 10^4^	-	2 × 10^4^	800
	100	5 × 10^8^	3 × 10^6^	2 × 10^4^	-	2 × 10^4^	
**59**	50	4 × 10^8^	2 × 10^6^	3 × 10^9^	2 × 10^9^	3 × 10^8^	1300
	100	5 × 10^8^	3 × 10^6^	3 × 10^9^	2 × 10^9^	3 × 10^8^	

Concn: concentration; MNC: maximum noncytotoxic concentration.

**Table 8:** Structural details and antiviral activity of compounds **61**–**64** (adapted from [105]).

	R’	R	Viral Infection Control (%)	
			1000 ppm	100 ppm
**61**	4-CH_3_O	D-glucopentyl	69	26
**62**	4-CH_3_O	D-xylobutyl	82	35
**63**	2-CH_3_	D-glucopentyl	59	22
**64**	2-CH_3_	D-xylobutyl	76	30
**Virazole**			100	100
